# An Analysis Based on Japonica Rice Root Characteristics and Crop Growth Under the Interaction of Irrigation and Nitrogen Methods

**DOI:** 10.3389/fpls.2022.890983

**Published:** 2022-06-28

**Authors:** Zhuoqian Wang, Yan Jia, Jinxu Fu, Zhaojun Qu, Xinpeng Wang, Detang Zou, Jingguo Wang, Hualong Liu, Hongliang Zheng, Jin Wang, Liang Yang, Huimin Xu, Hongwei Zhao

**Affiliations:** ^1^Key Laboratory of Germplasm Enhancement, Physiology and Ecology of Food Crops in Cold Region, Ministry of Education, Northeast Agriculture University, Harbin, China; ^2^Bei Da Huang Kenfeng Seed Limited Company, Harbin, China; ^3^Agricultural College, Northeast Agriculture University, Harbin, China; ^4^College of Arts and Sciences, Northeast Agriculture University, Harbin, China

**Keywords:** irrigation method, N split application, root physiological characteristics, crop growth, N use efficiency

## Abstract

Water shortages and nitrogen (N) fertilizer overuse limit japonica rice production in Northeastern China. The interactions between water-saving irrigation and nitrogen management on rice root and shoot growth is still our research focus. Here, japonica rice (DN425) was subjected to the irrigation methods W1 (flooding irrigation), W2 [mild alternate wetting and drying irrigation (AWD); −10 kPa], W3 (severe AWD; −30 kPa), and different N fertilizer ratios were applied in different growth stages, namely, N1 (6:3:1:0), N2 (5:3:1:1), and N3 (4:3:2:1). From jointing to full heading stages, the highest photosynthate production capacity and root activity were obtained under W1N2. AWD markedly affected the root system and resulted in root senescence at later growth stages. Grain yield and N utilization efficiency were closely and positively correlated with the relative water content, crop growth rate (CGR), leaf area duration (LAD), the increase rate of root length density, root surface area density, and root volume density (RVD) from the jointing to full heading stages. This positive correlation was also observed in the increased rate of root bleeding sap (RBS) under W1N2 and CGR under W2N3. From full heading to maturity stages, N2 could promote root growth, LAD, and CGR under AWD to a greater extent than those under the other treatments. Water use efficiency (WUE) and N uptake efficiency (NUpE) were both negatively associated with the decreased rate of RVD, root dry weight (RDW), and RBS. They were closely and positively correlated with the increased rate of RDW and CGR. Our results suggested that W2N2 treatment delayed root senescence, maintained leaf photosynthesis, optimized the crop growth rate from full heading to maturity stages, and improved grain yield. The optimal grain yield, WUE, and NUpE were achieved at the irrigation water amount and topdressing fertilizer ratio of 41.40–50.34 × 10^2^ and 31.20–34.83 kg ha^–1^, respectively.

## Introduction

The global rice planting area has reached 167 million hectares, and most of it is under flooding irrigation (Source: FA0, 2018 data). The current shortage of water resources has affected four billion people worldwide (Mekonnen and Hoekstra, 2016). By 2025, 20% of the total rice crop will experience a water deficit (Tuong and Bouman, 2003). Therefore, it is necessary to replace traditional flooding irrigation with more environmentally sustainable irrigation methods. Water-saving irrigation techniques for rice cultivation have developed rapidly in recent years. These include intermittent dry spells (Feng, 2007), alternating wetting and drying irrigation (AWD) (Lampayan et al., 2015), and combining shallow water depth with wetness and dryness (Mao, 2001). Studies have shown that water-saving irrigation methods improve water use efficiency. AWD is currently the most widely used water-saving technology in rice production (Carrijo et al., 2017; Ishfaq et al., 2020). As roots absorb water and nutrients from the soil, they can resist soil water deficits. The complex underground environment of heterogeneous soils has hindered root research in the field (Turner, 2018; Umburanas et al., 2019). Studies have shown that root dry weight, length density, and total absorption surface area increased under AWD (Chu et al., 2014). Rice is slightly more sensitive to water stress during the vegetative than the flowering period (Ouyang et al., 2020). AWD increases root biomass and deeper root distribution at the heading stage and enhances root activity in mid- to late-filling (Chu et al., 2016, 2018a).

Root and shoot biomass (Katsura et al., 2007), leaf area and leaf area duration (Niknejhad et al., 2013), and large grain storage capacity (Chu et al., 2016) increase rice grain yield, nitrogen, and water use efficiency. The balance between the root and shoot systems was maintained by specific physiological activities rather than by changing the biomass distribution ratio (Engels, 1994). Higher root dry weight accumulation maintains root activity, provides sufficient nutrients and water for the shoot, and promotes photosynthate accumulation in the shoot. The decline in dry weight accumulation in the shoot reduced organic matter transport from the shoot to the root and accelerated root senescence (Barbosa et al., 2013). Improving root activity, root bleeding sap, and photosynthetic potential during the reproductive stage of rice are essential for achieving a high grain yield (Xu et al., 2019; Chen et al., 2020). Moderate AWD(when soil water potential reached −15 kpa) can enhance rice root growth (Zhang et al., 2009; Fang et al., 2018), and good root traits contribute to aboveground biomass and grain yield (Palta and Yang, 2014). The crop growth rate reduced significantly from heading to maturity under severe AWD (Liang et al., 2016). Appropriately increasing the root-shoot ratio improves viability and ensures an adequate long-term water supply for photosynthesis when the soil water content is low. Numerous photosynthetic products are required to maintain larger root systems. Thus, an excessively high root-shoot ratio reduces the adaptability of grain yield to soil water potential (Sainju et al., 2017). AWD enables grain yield with maximum water saving and benefit-cost ratio (Song et al., 2018; Poddar et al., 2022).

Rice cultivars vary in terms of their fertilizer requirements during different growth periods. Applying nitrogen based on crop demand and adopting proper nitrogen fertilizer management can significantly improve N absorption, nitrogen use efficiency, and nitrogen harvest index (Wang et al., 2017a). The N^15^ tracer method revealed that nitrogen absorbed during early growth is used mainly in the vegetative period (Ata-Ul-Karim et al., 2017; Wang et al., 2017b). Excessive fertilizer application at the early growth stages reduce root length, surface area, volume, and CGR at the later growth stages (Moe et al., 2014). Excess or deficient nitrogen temporarily promotes early root growth. However, it also inhibits later root growth, reduces dry matter accumulation and grain yield, and hastens root death (Peng et al., 2012). NUE can be optimized by applying proper panicle and grain N fertilizer (Xiong et al., 2018), but too large N fertilizer topdressing ratio can prolong the slow-release effect of fertilizer, leading to the delayed population development and late maturity (Hu and Zhang, 2017). Therefore, reasonable topdressing N fertilizer can promote grain yield and root growth in the middle and later stages of growth (Deng et al., 2020). Water and nitrogen can interact to regulate grain yield and resource use efficiency in rice (Liu et al., 2014; Yang et al., 2017). Long-term fertilization and irrational irrigation can significantly inhibit rice N and water use efficiency (Peng et al., 2006). Appropriate water and nitrogen management after heading promote root growth in deep soil (Mahajan et al., 2012). The vigorous shoots could supply sufficient carbohydrates to roots, and active roots may enhance shoot growth by ensuring adequate water and N fertilizer supply (Ju et al., 2015; Bektas et al., 2016). When root dry weight decreases because of water or nitrogen deficiency, more photosynthate is allocated to the roots (Fageria et al., 2014). We hypothesize that AWD coupling with appropriate N application can balance the relationships between root-shoot to stabilize grain yield; however, the optimal treatment and its effect on the key growth period in rice need to be further studied.

This 2-year experiment investigated the effects of interactions between irrigation methods and split N applications on (1) root characteristics (root morphology, root dry weight, and root bleeding sap); (2) shoot characteristics (leaf area duration, relative water content, and crop growth rate); (3) grain yield, WUE, NUE; and (4) the relationship between root and shoot characteristics. The results of this study comprise an essential reference for soil water and nitrogen management of japonica rice production in Northeastern China.

## Materials and Methods

### Growth Conditions and Experimental Design

Field experiments were carried out in the Acheng Area experimental site of Northeast Agricultural University in Harbin, Heilongjiang Province, China (45°34′–45°46′N, 126°22′–126°50′E), during the growing seasons of 2019–2020. The average soil characteristics of soil samples (0–20 cm) in the experimental sites are shown in Supplementary Table 1. The mean temperature and precipitation during the rice-growing season at a weather station are presented in Supplementary Figure 1.

Dongnong 425 (DN425) is the experimental material, a japonica rice variety in the cold region of Northeastern China. The experimental design was a split block design. The irrigation treatments were the main plots and N split application treatments as the sub-plots. The plot area was 27 m^2^, and the plots were separated by plastic film inserted into the soil to a depth of 50 cm. Seedlings were raised in the seedbed on April 18, 2019 and April 20, 2020, and transplanted on May 24, 2019 and May 28, 2020, with the hole spacing of 30 cm × 10 cm and three plants per hill.

### Irrigation Methods and Nitrogen Split Applications

The irrigation methods consisted of the continuously flooding method (W1), mild alternate wetting and drying irrigation (W2), and severe alternate wetting and drying irrigation (W3) were conducted from 10 days after transplanting (10 DAT) to maturity. W1 treatment was continuously flooded with 3–5 cm water level in the plots until 7 days before harvest. In alternate wetting and drying irrigation (AWD), plots were not irrigated until the soil water potential is at –10 and –30 kPa (soil moisture content 0.175 and 0.131 g g^–1^, respectively) at a depth of 15–20 cm soil (supervised by Soil Science Research Institute, Chinese Academy of Sciences, Nanjing, China), tension meter readings recorded at 6:00, 12:00, and 18:00 every day, supplement water to each pot for W2 and W3, and record the irrigation amount, respectively. AWD started 7 days after transplanting until the critical water demand period when 1–3 cm was irrigated, and then AWD continued until 7 days before harvest (Supplementary Figure 2). Removable shelters sheltered the experiment field during rain, and the shelters were removed after rain. Diseases, insects, and weeds were strictly controlled during the entire growth period in both years.

N fertilizer was supplied as urea (46% N). K and P fertilizers were applied as Potassium sulfate (50% K_2_SO_4_) and Ammonium phosphate (46% HPO_4_, 18%NH_4_), respectively. In this case, the total pure N fertilizer was 150 kg ha^–1^ of N fertilizer, and different nitrogen fertilizer ratios were applied in different growth stages; three N split applications were used: (N1) 60% basal fertilizer, 30% tillering fertilizer, 10% panicle fertilizer; (N2) 50% basal fertilizer, 30% tillering fertilizer, 10% panicle fertilizer, 10% grain fertilizer; and (N3) 40% basal fertilizer, 30% tillering fertilizer, 20% panicle fertilizer, 10% grain fertilizer. Basal fertilizer was usually applied to the soil at pre-transplanting (1 DAT). Topdressing fertilizer was applied at tillering (7 DAT), panicle initiation, and initial spikelet differentiation (the appearance of luminous flower primordia at the tips of elongating primary rachis–branches), respectively. The zero-N application (N0) was the control, and the crop relied upon both local soil N supply and biological N fixation; Calcium phosphate (16% PO_4_) and Potassium sulfate (50% K_2_SO_4_) were applied at pre-transplanting (1 DAT).

### Measurement Items and Methods

#### Plant Dry Weight, Root Morphology, and Root Bleeding Sap

Plants were sampled at the jointing, full heading, and maturity stages, and three rice plants with the same growth vigor were selected. The whole plants were dug out by core sampling, the soil blocks of 0.2 m × 0.2 m × 0.25 m of each rice plant, and put into 70 mesh sieve bags (Yang et al., 2007). Soil blocks from the rhizosphere were pressure-washed with water and the entire root system was collected. The root samples were then washed with tap water to remove impurities. The plant samples were dried to a constant weight at 80°C and weighed between 20 DAT and maturity.

Roots were sampled at the jointing, full heading, and maturity stage. Five rice plants in growth vigor were selected. Sampling was performed as previously described (“Plant Dry Weight, Root Morphology and Root Bleeding Sap”). The cleaned root samples in each soil core were scanned with an Epson Perfection Expression 1680 scanner (Seiko Epson Corp., Tokyo, Japan). Photographs of the roots were taken and analyzed with WinRHIZO software (Regent Instruments Inc., Quebec City, QC, Canada) to obtain root morphology parameters (length, surface area, and volume) for each soil core. Root length density (RLD), root surface area density (RSAD), and root volume density (RVD) were calculated as follows:


(1) $$\text{RLD}\,\left(\mathrm{cm}\,{\mathrm{cm}}^{-3}\right)=\mathrm{Root}\,\mathrm{length}\,\mathrm{in}\,\mathrm{soil}\,\mathrm{core}\,/\,\mathrm{Soil}\,\mathrm{core}\,\mathrm{volume}$$

(2) $$\text{RSAD}\,\left({\text{cm}}^{2}\,{\text{cm}}^{-3}\right)=\mathrm{Root}\,\mathrm{surface}\,\mathrm{area}\,\mathrm{in}\,\mathrm{soil}\,\mathrm{core}\,/\,\mathrm{Soil}\,\mathrm{core}\,\mathrm{volume}\,$$

(3) $$\text{RVD}\,\left({\text{cm}}^{3}\,{\text{cm}}^{-3}\right)=\mathrm{Root}\,\mathrm{volume}\,\mathrm{in}\,\mathrm{soil}\,\mathrm{core}\,/\,\mathrm{Soil}\,\mathrm{core}\,\mathrm{volume}$$

At 18:00 h daily between 20 DAT and maturity, rice stems were cut 10 cm above soil level. Pre-weighed cotton wool was placed on the wounds and covered with polyethylene film. The cotton wool containing root bleeding sap was collected at 6:00 h the following day and reweighed. The bleeding intensity was calculated from the change in cotton wool weight (Liu et al., 2020). The assay was repeated in triplicate.

#### Leaf Area Duration and Relative Water Content

Leaf area duration was determined from the leaf area and photosynthetic duration as shown in Eq. 4 below (Chu et al., 2018b). It reflects the potential photosynthetic power of the plant. The foregoing sampling time was used in these analyses. The leaf area was measured with a leaf meter (LI–3000C, Li-Cor, Lincoln, NE, United States).


(4) $$\text{LAD}\,\left({\text{m}}^{2}\,{\text{m}}^{-2}\,\text{d}\right)=\left(\text{L}\,1+\text{L}\,2\right)\times \left(\text{t}\,2-\text{t}\,1\right)/2$$

where L_1_ and L_2_ were used to measure leaf areas two times.

Leaves were sampled at jointing, full heading, and maturity. Three rice plants equal in growth vigor were selected. Cut the leaves into about 1 cm segments and mix them well. Take about 0.5 g of leaves and 20 ml of deionized water and mix them. Vacuum the test tube to make the leaves sink into the bottom of the tube. After soaking for 12 h, take it out and dry the water on the blade surface. The samples were dried to a constant weight at 80°C. The assay was repeated in triplicate (Jensen et al., 2020). Furthermore, leaf relative water content (RWC) was calculated as follows:


(5) RWC(%)=[(FW-DW)/(TW-DW)]×100

where FW was the fresh weight of the leaf, TW was the turgid weight measured after floating the leaf 12 h under dim light, and DW was the dry weight.

#### Crop Growth Rate

The crop growth rate reflects the daily dry weight of the plant as shown in Eq. 6 below (Zhang et al., 2017). Sampling was performed as previously described.


(6) $$\text{CGR}\,\left(g\,m^{-2}\,{\text{d}}^{-1}\right)=\left(\text{W}\,1-\text{W}\,2\right)\,/\,\left(\text{t}\,2-\text{t}\,1\right)$$

where W1 and W2 were used to measure dry biomass of aboveground two times.

#### Water Use Efficiency and Nitrogen Use Efficiency

N uptake efficiency (NUpE) is the capacity of plant roots to acquire N from soil; N utilization efficiency (NUtE) is the capacity of resistance to stress in a range of N environments (Tegeder and Masclaux-Daubresse, 2018).


(7) $$\text{NUpE}\,\left(g\,g^{-1}\right)=\left({\text{TNU}}_{\text{N}}-{\text{TNU}}_{0}\right)/\mathrm{Total}\,N\,\mathrm{application}\,\mathrm{amount}$$

(8) $$\text{NUtE}\,\left(g\,g^{-1}\right)=\left({\text{GY}}_{\text{N}}-{\text{GY}}_{0}\right)/\left({\text{TNU}}_{\text{N}}-{\text{TNU}}_{0}\right)$$

(9) $$\text{WUE}\,\left(\mathrm{kg}\,m^{-3}\right)={\text{GY}}_{\text{N}}/\text{IA}\,\left(\text{Viets, 1962}\right)$$

where GY_*N*_ and GY_0_ are grain yields with and without N fertilization, respectively. TNU_*N*_ and TNU_0_ are the total N uptake by plants with and without N fertilization, respectively. IA is the total amount of irrigation water during plant growth.

### Calculations and Statistical Analysis

To test the differences among different treatments, data analysis of variance (ANOVA) was performed using the SPSS 20.0 (Chicago, IL, United States), and calculating the standard deviation of the means (three independent experiments). The statistical model included sources of variation due to year (Y), irrigation method (W), N split application (N), and the Y × W, Y × N, W × N, and Y × W × N interactions. The figures were generated with Orginpro2021b and Mathematica 9.0 software. Based on the least square method, binary quadratic regression equations were established to calculate the amounts of water and topdressing N needed to maximize relative parameters. The amounts of water and nitrogen corresponding to the optimal solution of each index in the confidence interval were obtained. The relationship between variables was evaluated by correlation and regression analysis and the combined interaction between irrigation and N application was evaluated by principal component analysis (PCA). Biplots constructed with each index were represented as symbols and traits by vectors from the origin (Di Rienzo et al., 2011).

## Results

### Effects of Irrigation Methods and Nitrogen Split Applications on Root Characteristics

#### Root Dry Weight

Irrigation methods and N split applications significantly affected RDW at the full heading stage (*P* < 0.01; Table 1). RDW decreased by 12.5 and 14.1% under W3 than under W1 and W2. The highest values of RDW were observed under W2N2 (457.8 g m^–2^ on average in both years). RDW were 8.5, 6.9, 6.9, and 4.1% greater under W1N2, W2N2, W1N3, and W2N3, respectively, than those under N1. RDW increased by 4.7 and 8.0% under N1 and N2 in W3, than under N3, respectively.

**TABLE 1:** Effect of the irrigation methods and N split applications on root dry weight (RDW).

Treatments		2019			2020		
		FS (g m^–2^)	JS-FS (g day^–1^)	FS-MS (g day^–1^)	FS (g m^–2^)	JS-FS (g day^–1^)	FS-MS (g day^–1^)
**Irrigation (W)**							
W1		430.2a	8.31b	2.56a	439.7a	7.81b	2.30a
W2		439.1a	9.55a	2.28a	446.6a	9.21a	2.30a
W3		377.5b	8.79ab	2.46a	383.6b	8.02b	2.53a
**N split (N)**							
N1		404.6b	7.54b	2.62a	410.0b	7.42b	2.73a
N2		430.1a	9.68a	2.46ab	440.2a	8.10b	2.47b
N3		412.1b	9.44a	2.21b	419.7b	9.52a	1.94c
**W × N**							
W1	N1	410.6cd	7.17e	2.32cd	416.6d	6.59e	2.44b
	N2	444.8ab	8.45c	2.58bc	459.1ab	7.68cd	2.53b
	N3	435.3ab	9.31b	2.79ab	443.3bc	9.16b	1.94cd
W2	N1	422.8bc	8.04d	2.62bc	429.8cd	8.12c	2.85a
	N2	453.6a	10.29a	2.41c	462.0a	8.91b	2.37b
	N3	440.8ab	10.31a	1.80e	448.1ab	10.60a	1.69d
W3	N1	380.5e	7.40e	2.93a	383.5f	7.54d	2.90a
	N2	391.8de	10.29a	2.40c	399.4e	7.73cd	2.52b
	N3	360.3f	8.69c	2.05de	367.8g	8.78b	2.19bc

***F value***	**Y**	**W**	**N**	**Y × W**	**Y × N**	**W × N**	**Y × W × N**

FS	ns	67.44**	3.74*	ns	ns	7.11**	ns
JS-FS	ns	7.34**	26.14**	4.37*	71.06**	19.90**	12.84**
FS-MS	ns	ns	18.74**	4.73*	5.59**	13.88**	4.62**

*Shown are mean values (n = 3).*

*JS, jointing stage; FS, full heading stage; MS, maturity stage.*

*JS-FS represents the increase rate of RDW from jointing to full heading stage, and FS-MS represents the decrease rate of RDW from full heading to maturity stage. Values of different letters are significantly different at P < 0.05.*

*, **F values significant at the P = 0.05 and P = 0.01 levels, respectively. ns means non-significant at the P = 0.05 level.

Irrigation methods and N split applications significantly affected the increase rate of RDW from jointing to the full heading stage (*P* < 0.01; Table 1). The rate increased by 14.1 and 10.4% under W2, than under W1 and W3, respectively. The rate was greater under W2N2, W2N3, and W3N2 than under the other treatments in 2019, while it was greater under W2N3 in 2020. The rate increased with increasing topdressing fertilizer ratio in all irrigation methods. N split applications significantly affected the decrease rate of RDW from full heading to maturity stage (*P* < 0.01; Table 1). The lowest values of the rate were observed under W2N3 (1.75 g day^–1^ on average in both years) and the highest value of the rate was observed under W3N1 (2.92 g day^–1^ on average in both years). The rate increased with increasing topdressing fertilizer ratio under W1 in 2019, which decreased with increasing topdressing fertilizer ratio under W2 and W3.

#### Root Morphology Parameters

**FIGURE 1:**
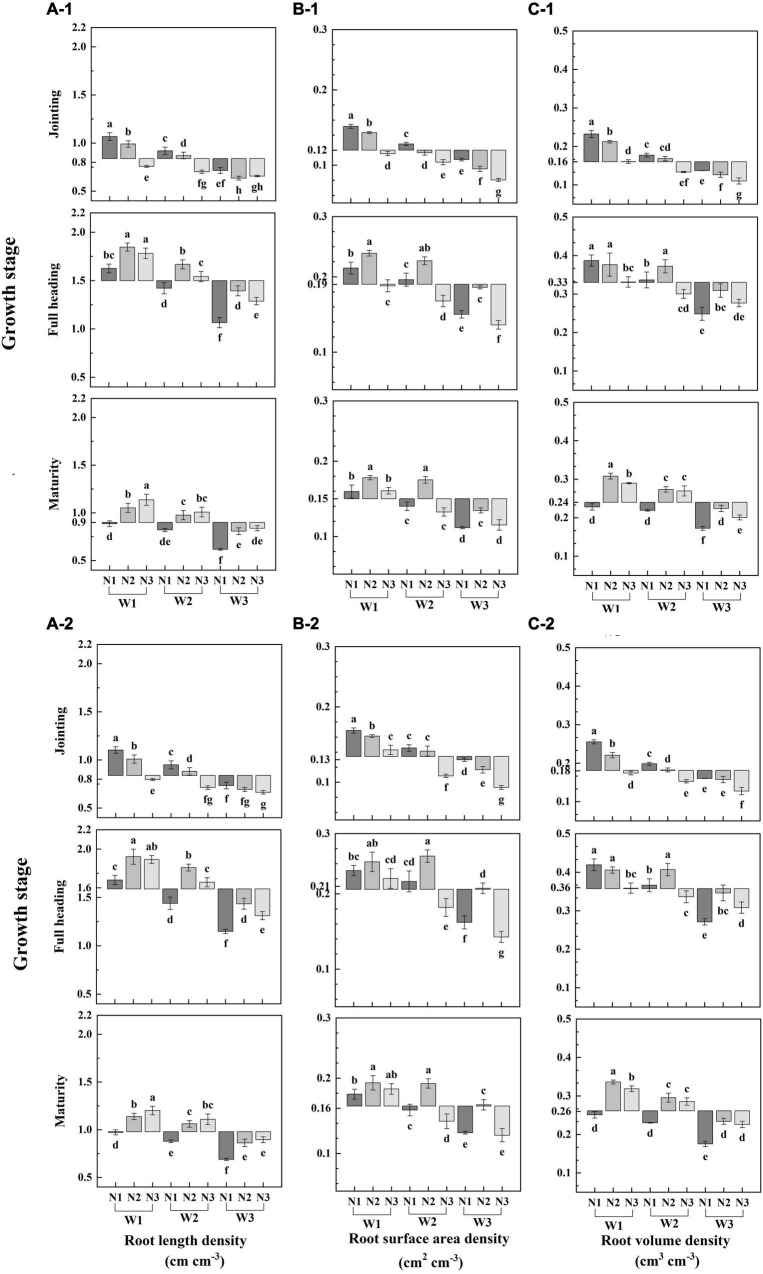
Effect of the irrigation methods and N split applications on total root length density, root surface area density and root volume density (**A–1,2, B–1,2, C–1,2**, in 2019 and 2020, respectively). Shown are the means values (*n* = 3). Values of different letters are significantly different at *P* < 0.05.

Irrigation methods significantly affected root length density, root surface area density, and root volume density (RLD, RSAD, and RVD) at each stage. At the jointing stage, all parameters were significantly lower under W2 and W3 than under W1. At full heading and maturity stages, RSAD and RVD were significantly lower only under W3 than under W1 and W2, but they had no differences between W1 and W2 (Figure 1).

N split application significantly affected RLD, RSAD, and RVD at each stage. The highest values of all parameters were observed under W1N1 at the jointing stage, followed by those under W1N2 (Figure 1). They were significantly greater under N1 and N2 than under N3 in all irrigation methods. At the full heading stage, RLD was greater under N2 and N3 in all irrigation methods than under N1; RLD under N2 was significantly greater than that under N3 in AWD. RSAD was significantly greater under W1N2 and W2N2 than under the other treatments. RSAD was significantly greater under N2 than under N1 and N3 in all irrigation methods; RSAD under N3 was lower than that under N1 in AWD. RVD was significantly greater under N2 than under the other treatments in AWD. At the maturity stage, RLD and RVD increased with increasing topdressing fertilizer ratio in all irrigation methods, but they showed no significant differences between N2 and N3 in AWD. RSADs under N2 were 19.4, 25.2, 19.8, and 19.5% greater than those under N1 and N3 in AWD, respectively. Hence, the irrigation method more strongly affected RVD at the jointing stage than after the full heading stage and RLD was more strongly affected at the full heading stage than at the jointing stage. The combination of N2 and AWD promoted the increase in root growth and elongation.

#### Root Bleeding Sap

**FIGURE 2:**
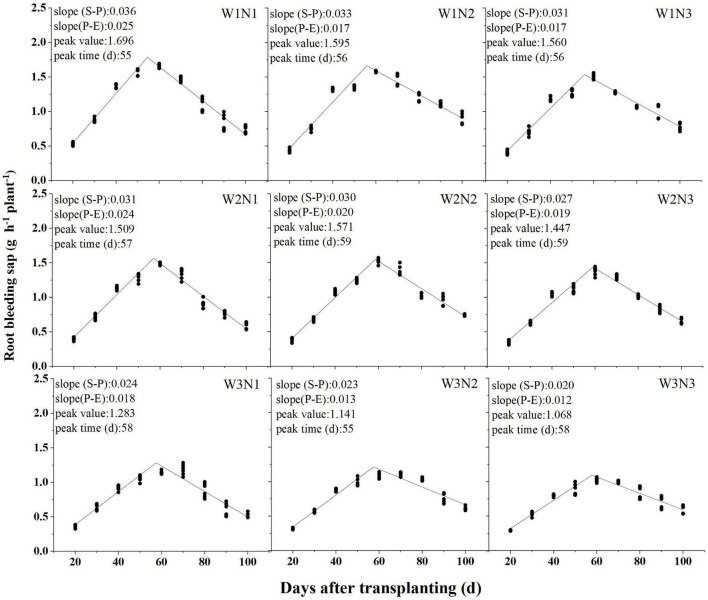
Effect of the irrigation methods and N split application on root bleeding sap. Shown are the means values (*N* = 6). The linear regressions were analyzed by pooling out data for 2 years. Numbers on the top are slope of the starting point to the peak slope (S-P). Numbers on the middle are absolute value slope of the peak to the ending point of the line slope (P-E). Numbers on the bottom are the time of piecewise linearity.

According to the piecewise linear function (Figure 2), the highest value of RBS was observed at 55 days after transplanting (DAT). RBS appeared both at 58 DAT under W2N1 and W3N1, it appeared ∼2 days later under N2 than N1. Under the same N split application, the highest value of RBS was observed under W1 but significantly decreased under W2 and W3. Furthermore, RBS was 10.6 and 9.4% greater under N1 and N2, respectively, than under N3. RBS was greater under N2 than N3 in AWD. The maximum increase rate of RBS was observed from 20 DAT to the maximum value under W1, which were 5.4 and 27.2% less under W2 and W3. The increase rate was greater under N1 and N2 than under N3 in all irrigation methods. The irrigation method more strongly affected RBS at the jointing stage than after the full heading stage. There were no differences between W1 and W2 in terms of the decrease rate of RBS. Compared with N1, the rate decreased with increasing topdressing fertilizer ratio in all irrigation methods.

#### Root-Shoot Ratio

Irrigation methods significantly affected R/S at each stage (*P* < 0.01; Table 2). Compared with W1, R/S was 10.9 and 13.6% greater under W2 and W3 at jointing, 8.7 and 7.5% at full heading, and 12.4 and 10.8% at maturity, respectively. At the jointing and full heading stages, R/S did not significantly differ between W2 and W3. Under all water and nitrogen treatments, R/S was greater under W2N2 and W3N2 than under the other treatments at the jointing stage. At full heading and maturity stages, the highest values of R/S were observed under W2N3. Compared with W1N2, R/S was 12.8, 8.6, and 8.7% greater under W2N2 from jointing to maturity stages, respectively.

**TABLE 2:** Effect of the irrigation methods and N split applications on the root-shoot ratio (R/S).

Treatments		2019			2020		
		JS	FS	MS	JS	FS	MS
**Irrigation (W)**							
W1		0.48b	0.52b	0.22c	0.49b	0.53b	0.22b
W2		0.55a	0.57a	0.25a	0.54a	0.58a	0.25a
W3		0.56a	0.57a	0.23b	0.56a	0.56a	0.22b
**N split (N)**							
N1		0.52ab	0.53b	0.23ab	0.52b	0.54b	0.22b
N2		0.56a	0.55b	0.23b	0.57a	0.56b	0.22b
N3		0.51b	0.60a	0.24a	0.50b	0.57a	0.23a
**W × N**							
W1	N1	0.49d	0.49e	0.22cd	0.47f	0.51f	0.21d
	N2	0.49d	0.52d	0.21d	0.53d	0.53ef	0.22cd
	N3	0.46e	0.55bc	0.22bcd	0.47f	0.55de	0.23bc
W2	N1	0.53bc	0.54cd	0.25a	0.55c	0.55cd	0.24b
	N2	0.59a	0.56bc	0.24b	0.58b	0.58ab	0.24b
	N3	0.51cd	0.61a	0.27a	0.49e	0.60a	0.27a
W3	N1	0.55b	0.58ab	0.23bc	0.52d	0.56cd	0.22cd
	N2	0.59a	0.56bc	0.22bcd	0.62a	0.57bc	0.21d
	N3	0.55b	0.58ab	0.23bc	0.53d	0.55cd	0.21d

***F value***	**Y**	**W**	**N**	**Y × W**	**Y × N**	**W × N**	**Y × W × N**

JS	ns	26.05**	13.21**	ns	4.09*	5.48**	ns
FS	ns	22.99**	6.68**	3.74*	3.61*	8.26**	ns
MS	ns	37.87**	ns	3.39*	Ns	5.93**	ns

*Shown are mean values (n = 3).*

*Values of different letters are significantly different at P < 0.05.*

*, **F values significant at the P = 0.05 and P = 0.01 levels, respectively. ns means non-significant at the P = 0.05 level.

### Effects of Irrigation Methods and Nitrogen Split Applications on Crop Growth

#### Growth Period

On average, the growth days were 1.5 days shorter under W3 than W1 from jointing to full heading stages (Table 3). However, the growth days were prolonged under W2 and W3 from full heading to maturity, which were 2.7 and 2 days on average, respectively. Compared with N1, the growth days were shortened with increasing topdressing fertilizer ratio from jointing to full heading stages in all irrigation methods. By contrast, the growth days were prolonged from full heading to maturity stages.

**TABLE 3:** Effect of the irrigation methods and N split applications on days of the growth period (d).

Treatment		2019		2020	
		JS-FS	FS-MS	JS-FS	FS-MS
W1	N1	29	39	31	41
	N2	27	39	30	42
	N3	27	41	28	44
W2	N1	29	41	30	42
	N2	27	43	29	46
	N3	27	43	27	47
W3	N1	28	41	30	42
	N2	26	44	27	44
	N3	26	43	26	44

#### Leaf Area Duration

Irrigation methods and N split applications significantly affected LAD at each stage (*P* < 0.05; Table 4). The LAD was lower in 2019 than in 2020 at each stage. Compared with W1, the LAD decreased under W2 and W3 from jointing to full heading and from full heading to maturity stages, by 10.8% and 26.7, 8.3, and 14.8%, respectively. The highest value of LAD was observed under W1N1 from jointing to full heading stages in 2019; it appeared under W1N1 and W1N2 in 2020. The LAD significantly decreased with increasing topdressing fertilizer ratio in AWD. The highest value of LAD was observed under W1N3 from full heading to maturity stages; and it gradually increased with topdressing fertilizer ratio under W1. The LAD under N2 were 15.5, 3.2, 13.3, and 4.3% lower than those under N1 and N3 in AWD, respectively.

**TABLE 4:** Effect of the irrigation methods and N split applications on leaf area duration (LAD).

Treatments		2019			2020		
		JS-HS	FS-MS		FS-HS	HS-MS	
**Irrigation (W)**							
W1		185.33a	227.27a		204.23a	249.73a	
W2		167.45b	217.13a		180.00b	240.24a	
W3		135.33c	186.29b		150.25c	203.19b	
**N split (N)**							
N1		171.17a	192.27b		187.99a	212.25b	
N2		162.40a	221.13a		182.35a	242.17a	
N3		154.54a	217.38a		164.14a	238.74a]	
**W × N**							
W1	N1	192.68a	211.36d		209.61a	231.04d	
	N2	185.25b	232.30b		211.74a	256.20b	
	N3	178.06c	238.15a		191.33b	261.95a	
W2	N1	177.49c	197.02e		190.60b	215.11e	
	N2	167.97d	230.94b		186.28c	256.76b	
	N3	156.88e	223.45c		163.13d	248.87c	
W3	N1	143.36f	168.15g		163.75d	190.61g	
	N2	133.97g	200.17e		149.04e	213.55e	
	N3	128.67h	190.55f		137.97f	205.41f	

***F value***	**Y**	**W**	**N**	**Y × W**	**Y × N**	**W × N**	**Y × W × N**

JS-FS	5.75*	80.83**	3.38*	18.89**	51.67**	23.25**	9.00**
FS-MS	10.31**	29.58**	9.08**	5.78**	ns	18.03**	3.91*

*Shown are mean values (n = 3) (m^2^m^–2^day).*

*Values of different letters are significantly different at P < 0.05.*

*, **F values significant at the P = 0.05 and P = 0.01 levels, respectively. ns means non-significant at the P = 0.05 level.

#### Leaf Relative Water Content

Irrigation methods and N split applications significantly affected RWC at each stage (*P* < 0.01; Table 5). At the jointing stage, RWCs under W2 were 4.9 and 10.6% greater than those under W1 and W3, respectively. At full heading and maturity stages, RWCs under both W1 and W2 were greater than those under W3, respectively. Under all water and nitrogen treatments, RWCs were greater under W2N1 and W2N2 than under other treatments at the jointing stage. At the full heading stage, RWC was greater under N2 than N1 and N3 in all irrigation methods. At the maturity stage, RWC was greater under N2 than under other treatments in W1, the RWCs under N1 were 7.0, 4.4, 9.0, and 5.8% lower than those under N2 and N3 in AWD, respectively.

**TABLE 5:** Effect of the irrigation methods and N split applications on leaf relative water content (RWC).

Treatments		2019			2020		
		JS	HS	MS	JS	HS	MS
**Irrigation (W)**							
W1		73.54ab	85.56a	86.07a	72.48ab	85.94a	85.74a
W2		77.78a	86.70a	87.17a	75.70a	83.97ab	89.54a
W3		69.81b	79.79b	79.38b	67.96b	78.38b	78.12b
**N split (N)**							
N1		76.74a	83.28b	80.71b	75.15a	82.34b	80.17b
N2		75.32a	88.90a	87.85a	74.03a	87.84a	88.38a
N3		69.06b	79.87b	84.05ab	66.96b	78.12b	84.85ab
**W × N**							
W1	N1	74.05b	85.28bc	82.36bc	74.19b	85.69ab	80.95d
	N2	72.26bc	89.25ab	91.55a	71.29bcd	89.50a	90.24b
	N3	74.31b	82.15c	84.30b	71.97bc	82.64b	86.01c
W2	N1	81.78a	86.60b	84.25b	77.89a	84.34b	85.56c
	N2	82.23a	91.99a	89.27a	80.32a	89.62	93.31a
	N3	69.32d	81.51cd	87.98a	68.87d	77.96c	89.76b
W3	N1	74.40b	77.96de	75.52d	73.37bc	76.99cd	74.01f
	N2	71.45cd	85.47bc	82.74bc	70.47cd	84.40b	81.58d
	N3	63.56e	75.94e	79.88c	60.03e	73.76d	78.76e

***F value***	**Y**	**W**	**N**	**Y × W**	**Y × N**	**W × N**	**Y × W × N**

JS	Ns	12.79**	16.53**	ns	ns	150.35**	ns
HS	Ns	13.61**	31.75**	7.55**	ns	7.31**	ns
MS	Ns	36.27**	13.56**	19.98**	ns	7.04**	ns

*Shown are mean values (n = 3) (%).*

*Values of different letters are significantly different at P < 0.05.*

*, **F values significant at the P = 0.05 and P = 0.01 levels, respectively. ns means non-significant at the P = 0.05 level.

#### Crop Growth Rate

Irrigation methods and N split applications significantly affected CGR (*P* < 0.05; Table 6). From jointing to full heading stages, CGR under W2 was 10.7 and 8.0% greater than under W1 and W3, respectively. The highest value of CGR was observed under W2N2 and W2N3. The CGR under N1 was 12.6 and 15.6% lower than under N2 and N3 in W1, respectively. The CGR under N2 was significantly greater than under the other treatments in AWD in 2019; it gradually increased with topdressing fertilizer ratio under all irrigation methods in 2020. From full heading to maturity stages, CGR under W1 was 23.9 and 30.9% greater than under W2 and W3, respectively. The highest value of CGR was observed under W1N2. The CGR increased under N2 and N3 in all irrigation methods, than that under N1.

**TABLE 6:** Effect of the irrigation methods and N split applications on crop growth rate (CGR).

Treatments		2019		2020	
		JS-FS	FS-MS	JS-FS	FS-MS
**Irrigation (W)**					
W1		23.24b	15.31a	21.28b	15.14a
W2		25.33a	11.63b	24.42a	11.47b
W3		23.30b	9.96b	22.35ab	10.99b
**N split (N)**					
N1		21.56b	10.24b	20.55c	11.09b
N2		25.94a	13.60a	22.84b	13.42a
N3		24.36a	13.07a	24.66a	13.09a
**W × N**					
W1	N1	21.99e	13.65c	18.19g	14.59a
	N2	24.01c	16.58a	21.98e	15.36a
	N3	23.71cd	15.71b	23.67bc	15.45a
W2	N1	22.76de	8.62e	22.98cd	9.64d
	N2	27.45a	13.25c	24.14b	12.79b
	N3	25.77 b	13.03c	26.13a	11.99c
W3	N1	19.93f	8.46e	20.46f	9.05d
	N2	26.37ab	10.95d	22.39de	12.10b
	N3	23.59cd	10.46d	24.19b	11.83bc

***F value***	**Y**	**W**	**N**	**Y × W**	**Y × N**	**W × N**	**Y × W × N**

JS-HS	ns	7.23**	20.87**	ns	17.01**	ns	3.63*
HS-MS	ns	45.55**	8.14**	3.86*	ns	3.50*	ns

*Shown are mean values (n = 3) (g m^–2^ day^–1^).*

*Values of different letters are significantly different at P < 0.05.*

*, **F values significant at the P = 0.05 and P = 0.01 levels, respectively. ns means non-significant at the P = 0.05 level.

### Effects of Irrigation Methods and Nitrogen Split Applications on Grain Yield, Water Use Efficiency, and Nitrogen Use Efficiency (Nitrogen Uptake Efficiency and Nitrogen Utilization Efficiency)

Irrigation methods and N split applications significantly affected rice grain yield (*P* < 0.01, Table 7). Grain yield decreased by 9.3 and 21.3% under W2 and W3, respectively, than under W1. The highest value of grain yield was observed under W1N2 (8,578.0 kg ha^–1^ on average in both years), followed by those under W2N2 (decreased by 5.4%). Grain yield was significantly greater under N3 than under N1 in AWD. Irrigation methods significantly affected rice WUE (*P* < 0.01, Table 7). WUE was greater under W2 (25.9%) and W3 (27.6%) than under W1. The highest value of WUE was observed under W2N2 (1.91 kg m^–3^ on average in both years). Compared with N1, WUE increased by 19.1, 9.5, 12.3, and 9.8% under N2 and N3 in AWD, respectively. Irrigation methods significantly affected rice NUtE (*P* < 0.05, Table 7). NUtE decreased under W3 than under W1 and W2, by 10.5 and 10.8%. N split applications affected rice NUpE and NUtE (*P* < 0.01, Table 7). NUpE under N2 were 34.1, 14.7, 27.1, and 11.2% greater than those under N1 and N3 in AWD, respectively. NUtE under N2 were 15.5, 25.5, 17.6, and 11.4% greater than those under N1 and N3 in W1 and W2, respectively.

**TABLE 7:** Effect of the irrigation methods and N split applications on grain yield (GY) water use efficiency (WUE), IA (irrigation amount), N uptake efficiency (NUpE), and N utilization efficiency (NUtE).

Treatments		2019					2020				
		GY (kg ha^–1^)	WUE (kg m^–3^)	IA (×10^2^ kg ha^–1^)	NUpE (g g^–1^)	NUtE (g g^–1^)	GY (kg ha^–1^)	WUE (kg m^–3^)	IA (×10^2^ kg ha^–1^)	NUpE (g g^–1^)	NUtE (g g^–1^)
**Irrigation (W)**											
W1		7778.0a	1.27b	61.14a	0.42a	45.2a	7830.8a	1.24b	63.01a	0.42a	50.40a
W2		7064.9b	1.73a	42.75b	0.43a	47.5a	7089.6b	1.66a	44.65b	0.43a	48.16ab
W3		6073.2c	1.75a	35.40c	0.43a	42.1a	6211.2c	1.73a	36.53c	0.44a	43.31b
**N split (N)**											
N1		6319.5c	1.47b	44.64a	0.36c	42.30b	6378.0c	1.42a	46.69a	0.36c	45.23b
N2		77735.0a	1.71a	47.55a	0.48a	49.75a	7756.8a	1.66a	49.22a	0.45b	51.47a
N3		6861.6b	1.56b	47.11a	0.43b	42.67b	6996.8b	1.55a	48.29a	0.49a	45.14b
**W × N**											
W1	N1	7262.1cd	1.24f	58.60b	0.38cd	44.06bc	7304.7cd	1.20f	60.77b	0.36c	49.54bc
	N2	8542.7a	1.36e	62.77a	0.44bc	53.26a	8613.2a	1.34e	64.50a	0.46b	57.42a
	N3	7529.1c	1.21f	62.06a	0.43c	38.29d	7574.4c	1.19f	63.77a	0.44b	44.27de
W2	N1	6132.4f	1.56d	41.12d	0.34d	42.75bcd	6203.8f	1.48d	43.85d	0.34c	44.44de
	N2	8080.4b	1.94a	43.08c	0.52a	53.42a	7993.2b	1.82a	45.55c	0.51a	52.47b
	N3	6982.0d	1.68c	44.06c	0.44bc	46.20b	7071.8d	1.68b	44.55d	0.44b	47.57cd
W3	N1	5563.9g	1.63cd	34.20f	0.36d	40.08cd	5625.5g	1.59c	35.45g	0.37c	41.71e
	N2	6582.0e	1.84b	36.80e	0.50ab	42.57bcd	6663.9e	1.83a	37.60e	0.50a	44.53de
	N3	6073.6f	1.78b	35.20f	0.42c	43.54bc	6344.3ef	1.79a	36.55f	0.46b	43.58de

***F value***		**Y**	**W**	**N**		**Y × W**	**Y × N**		**W × N**		**Y × W × N**

GY		ns	20.42**	10.71**		ns	ns		9.93**		ns
WUE		ns	59.09**	ns		ns	ns		13.42**		ns
IA		ns	808.64**	ns		ns	ns		8.61**		ns
NUpE		ns	ns	78.12**		ns	ns		5.32**		ns
NUtE		ns	3.33*	6.67**		ns	ns		ns		ns

*Shown are mean values (n = 3).*

*Values of different letters are significantly different at P < 0.05.*

*, **F values significant at the P = 0.05 and P = 0.01 levels, respectively. ns means non-significant at the P = 0.05 level.

### Coupling Effect of Irrigation and Nitrogen Application on Rice Grain Yield, Water Use Efficiency, and Nitrogen Uptake Efficiency

The results showed that water and nitrogen inputs had a highly significant influence on the dependent variables (*P* < 0.01; except for NUtE, which was not considered in the following analysis). The amounts of irrigation and topdressing N fertilizer corresponding to the maximum value were shown in Table 8. The maximum grain yield was 8,672.4 kg ha^–1^ when 5,630 kg ha^–1^ of irrigation water and 31.8 kg ha^–1^ of topdressing N fertilizer were applied. The maximum WUE was 1.914 kg/kg when 3,200 kg ha^–1^ of irrigation water and 33.6 kg ha^–1^ of topdressing N fertilizer were applied. The maximum NUpE was 0.524 g g^–1^ when 5,000 kg ha^–1^ of irrigation water and 33.8 kg ha^–1^ of topdressing N fertilizer were used.

**TABLE 8:** Regression equation between water and nitrogen input and GY, WUE, NUpE, and NUtE.

Response variable Y	Regression equation	R^2^	Y max	IA (×10^2^ kg ha^–1^)	N_*t*_ (kg ha^–1^)
GY/Y_1_ (kg ha^–1^)	Y_1_ = −9109.936 + 457.058*I* + 310.290*N* −3.959*I*^2^−4.546*N ^2^*−0.375*IN*	0.93**	8672.40	56.28	31.78
WUE/Y_2_ (kg m^–3^)	Y_2_ = 0.1086 + 4.081 × 10^–2^*I* + 6.864 × 10^–2^*N* −5.7 × 10^–4^*I*^2^−9.6 × 10^–4^*N ^2^*−1.3 × 10^–4^*IN*	0.97**	1.92	34.89	33.62
NUpE/Y_3_ (g g^–1^)	Y_3_ = −0.107 + 6.82 × 10^–3^*I* + 2.7 × 10^–2^*N* −7.316 × 10^–5^*I*^2^−4.127 × 10^–4^*N*^2^−6.6 × 10^–6^*IN*	0.87**	0.48	45.15	32.36
NUtE/Y_4_ (g g^–1^)	Y4 = −24.616 + 1.627*I* + 2.178*N* −1.1 × 10^–2^*I*^2^−2.8 × 10^–2^*N*^2^−1.1 × 10^–2^*IN*	0.53*^ns^*	54.00	60.40	27.00

*The maximum GY, WUE, NUpE, and NUtE with the amount of irrigation and topdressing N fertilizer.*

*I and N mean irrigation amount and topdressing N fertilizer, respectively.*

**P < 0.01. ns, non-significant at the P = 0.05 level.

**FIGURE 3:**
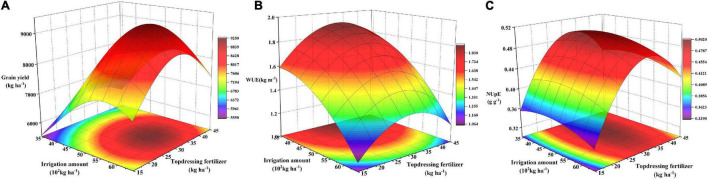
Relationships among water and nitrogen input and grain yield **(A)**, water use efficiency (WUE, **B)**, and N uptake efficiency (NUDE, **C)** of japonica rica.

Grain yield, WUE, and NUpE could not be maximized simultaneously, so the water and nitrogen fertilizer input should be further analyzed to determine the best coupling treatments. The coupling effects of irrigation and N application on the rice grain yield, WUE, and NUpE in the 2-year experiment exhibited a downward convex shape. Grain yield, WUE, and NUpE in 95, 90, 85, and 80% acceptable regions were analyzed and presented that they could be simultaneously obtained within the 90% acceptable range, and their ranges were similar (Figure 3). When all the factors in the 2-year experiment were comprehensively considered, the optimal grain yield, WUE, and NUpE were obtained with the irrigation water and topdressing fertilizer amount of 41.40–50.34 × 102 and 31.20–34.83 kg ha^–1^, respectively.

### Relationships Among Root Characteristics and Crop Growth

**FIGURE 4:**
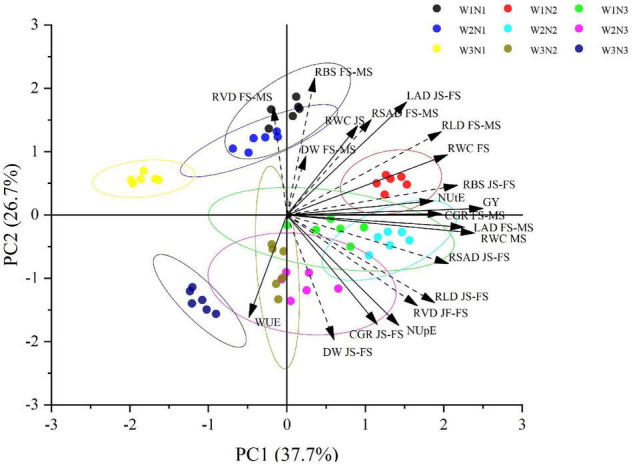
The relationships between root characteristics and crop growth under irrigation and N split application in japonica rice. Dashed vectors represent the increase rate and the decline rate of root characteristics from jointing to full heading stage (JS-FS) and full heading to maturity stage (FS-MS), respectively. RLD, root length density; RSAD, root surface area density; RVD, root volume density; DW, root dry weight; RBS, root bleeding sap; LAD, leaf area duration; CGR, crop growth rate; RWC, root relative water content; GY, grain yield; WUE, water use efficiency; NUpE, N uptake efficiency; NUtE, N utilization efficiency.

Principal component analyses were used to evaluate the relationships among root and shoot growth under different treatments. The first analysis involved crop growth rate and leaf area duration from the full heading to maturity stages and the increased rate of root bleeding sap from the jointing to full heading stages (Figure 4). PC1 explained the variation in grain yield and NUtE, which were closely and positively correlated with RWC, CGR, LAD, and the increased rate of RLD, RSAD, and RVD from jointing to full heading stages. PC2 mainly involved RDW, the decreased rate of RBS and RVD; It also explained the variations in WUE and NUpE, which were in the negative direction of the axis. They were closely and positively correlated with the increased rate of RDW and CGR. By contrast, WUE and NUpE were negatively associated with the decreased rate of RVD, RDW, and RBS from full heading to maturity stages.

The factors of W1N2 were its high RWC at the full heading stage, increase rate of root bleeding sap, and the decreased rate of RLD. The factors of W2N2 were its high increase rate of RLD, RSAD, and RVD and the decreased rate of crop growth and leaf area duration. PC2 discriminated among topdressing fertilizer ratios in all irrigation methods, with the data of N1 toward positive values and the data of N2 and N3 toward negative values. This trend indicated that the higher the topdressing fertilizer ratio, the higher the increase rate of root characteristics. Considering the W × N combinations, PCA showed that W3N2 could alleviate the adverse effects of W3 on rice indices, but coupled N3 treatment under W1 and W2 had no significant positive effect. As a result of these trends, grain yield was explained by the increased rate of root bleeding sap under W1N2 (GY = 9,797.26–4.03 × 10^4^ RBS, R^2^ = 0.75), and NUpE was more strongly related to CGR (GY = 1.106–0.026 CGR, R^2^ = 0.78) than to the increase rate of RVD (GY = 0.536–0.149 RVD, R^2^ = 0.70) under W2N3. NUtE was more strongly related to the decreased rate of crop growth (GY = 91.39–2.26 CGR, R^2^ = 0.78) than to the increased rate of root bleeding sap (GY = 81.02–849.2 RBS, R^2^ = 0.59) under W1N2.

## Discussion

### Effects of Irrigation Methods and Nitrogen Split Applications on Root Characteristics and Crop Growth

This manuscript attempted to gain insights into the water-nitrogen relationships, and thus optimize irrigation methods and N split applications to confirm whether the reasonable N fertilizer application can be used to balance the relationships of root-shoot under AWD. Water-saving irrigation improves root activity and establishes good root morphology more effectively than flooding irrigation (Alou et al., 2018; Song et al., 2018). However, total root length, surface area, and volume significantly decreased under severe water-saving irrigation, which was not conducive to root nutrient absorption at the later growth stages (Narayanan and Prasad, 2014). The irrigation method more strongly influences root volume density at full heading at jointing than it does after the full heading stage, and its effect on root length density was more strongly at full heading than it does at jointing (Figure 1).

The piecewise linear function showed that the time of maximum root bleeding sap did not correspond with the shoot growth period (Figure 2). Shortening the rice growth period under AWD created an imbalance in root and shoot growth, which caused the shoot to reach its peak growth first. Increasing the amount of topdressing fertilizer can balance the overall growth of plants. From jointing to full heading stage, W2 could increase root dry weight and crop growth rate, and the root-shoot ratio was greater than that of W1, indicating that W2 treatment could promote the whole plant growth. At this stage, the increase rate of root dry weight and crop growth rate was greater under N3 in all irrigation methods, but it decreased significant leaf area duration than under the other treatments; a similar conclusion was also presented by Santiago-Arenas et al. (2019). From the full heading to maturity stage, the decrease rate of root dry weight and root activity was delayed under W1 with an increasing ratio of topdressing fertilizer, but to what extent of the increase needs to be further studied. Root growth and elongation, leaf photosynthetic capacity, and adaptability to AWD were enhanced under AWD couple N2, while the potential decline in N3 treatment may be caused by very few basal fertilizer amounts, and the whole rice plant can still absorb basal fertilizer from the soil after full heading (Moe et al., 2014).

A decrease in root physiological activity accelerates leaf senescence and shortens grain filling (Yang et al., 2012). The growth period was prolonged under AWD from full heading to maturity stages (Table 3). The grain filling period can be prolonged by increasing topdressing fertilizer ratio under W2 and W3, which can provide more environmental resources for rice (Yang et al., 2007). Consequently, reproductive growth was shortened and there was less time for the roots to transport nutrients and water to the shoots in the later growth stages. This discrepancy could result in redundant root growth, photosynthate overconsumption, an imbalance between root and shoot growth, and reduced grain yield (Ma et al., 2010).

Although water and N interact with each other and have a coupled effect on rice growth (Reis, 2018), it remained to be determined how N2 and N3 regulate root and shoot traits in AWD. AWD irrigation along with N application increases the root and shoot growth (Pascual and Wang, 2017). Good rice root morphology helps regulate the interaction between irrigation and N fertilizer. Under AWD, the root system effectively absorbs nutrients and water and maintains plant growth (Comas et al., 2013). Maximum root dry weight appeared at full heading under W2N2 (Table 1). Root bleeding sap was positively correlated with photosynthetic rate during grain filling (Meng et al., 2018). Root length and volume density were positively correlated with shoot biomass at heading and maturity. W1N2 and W2N2 maintained the balance between root and shoot growth and improved foliar photosynthetic capacity and AWD adaptability.

### Effects of Irrigation Methods and Nitrogen Split Applications on Grain Yield, Water Use Efficiency, Nitrogen Uptake Efficiency, and Nitrogen Utilization Efficiency

Water consumption was 25–30% lower under AWD than flooding irrigation (Rahman and Bulbul, 2015). Nevertheless, conclusions vary in terms of the impact of AWD on grain yield (Jabran et al., 2015; Carrijo et al., 2017; Kumar et al., 2017; Yang et al., 2017), which is related to the severity of the applied drying cycle and different operational measures in different regions (Price et al., 2013; Ogirigbo and Durojaye, 2020). Further studies have revealed a win-win relationship between WUE and yield at the appropriate irrigation level (Yang et al., 2017). Indeed, we found that rice grain yield was lower under W2 and W3, than in W1. Here, grain yield was significantly greater under W1N2 and W2N2 than in the other treatments. Grain yield was 9.4 and 6.0% greater under W2N2 than W1N1 and W1N3, respectively, and it was greater under W3N2 than W2N1 (6.9%) (Table 7), indicating that the effect of N fertilizer on grain yield reduced when the topdressing N fertilizer reached a certain level. The interaction effect showed that the effect of the irrigation method on grain yield under AWD was greater than that of nitrogen application. WUE was significantly greater under W2N2 and W2N3 than under the other treatments (Table 7). Nitrogen fertilizer application at different growth stages can improve NUE (Agyin-Birikorang et al., 2017; Shan et al., 2017). In the present study, NUpE was not affected by irrigation methods and could increase with increasing topdressing fertilizer ratio under all irrigation methods.

Based on the quadratic models, the results showed that water and nitrogen inputs had a highly significant influence on the dependent variables, except for NUtE, which was not considered in the following analysis (Table 8). When all the factors in the 2-year experiment were comprehensively considered, the optimal grain yield, WUE, and NUpE were obtained with the irrigation water and topdressing fertilizer amount of 41.40–50.34 × 10^2^ and 31.20–34.83 kg ha^–1^, respectively. It reported that AWD has the potential to increase yield when moderate AWD maintains soil water potential ≥–20 kPa, it can ensure soil moisture for optimal growth, which is consistent with the proposed W2 treatment coupled with N2 and can effectively adjust the grain yield and use efficiency (Norton et al., 2017; Carrijo et al., 2018).

Principal component analyses were used to evaluate the relationships between root and shoot growth under different treatments (Figure 4). Grain yield and NUtE were closely and positively correlated with relative water content, crop growth rate, leaf area duration, increase rate of root morphology parameters, and root activity from the jointing to full heading stages. WUE and NUpE were negatively associated with the decline rate of root volume density, root dry weight, and root bleeding sap. They were closely and positively correlated with the increased rate of root dry weight and crop growth rate. The increased rates of root bleeding sap and root volume density were key indicators (Figure 4). The increase in the root bleeding intensity from the jointing to full heading stages could increase the relative water content of leaves at the full heading stage and improve their drought resistance. Increasing the rate of root dry weight could promote the growth of the aboveground population. Grain yield was explained by the increased rate of root bleeding sap under W1N2, and NUtE was more strongly related to the increased rate of root bleeding sap under W1N2. The factors of W2N2 were its high increase rates of root length density, root surface area density, and root volume density and the decrease rate of crop growth and leaf area duration. Considering the W × N combinations, PCA showed that W3N2 could alleviate the adverse effects of W3 on rice indices, but coupled N3 treatment under W1 and W2 had no significant positive effect.

## Conclusion

The present study demonstrated the significant influences of irrigation methods and N split applications on rice root characteristics and crop growth. Grain yield and NUtE were closely and positively correlated with the increased rate of root length density, root surface area density, root volume density, and root bleeding sap from the jointing to full heading stages. Shortening the rice growth period under AWD created an imbalance in root and shoot growth and caused the shoot to reach its peak growth first. The root-shoot growth balance was maintained under W2N2 and W2N3. WUE and NUpE were negatively associated with the decreased rate of root volume density, root dry weight, and root bleeding sap from the full heading to maturity stages. They were closely and positively correlated with the increased rate of root dry weight and crop growth rate. Under AWD, reproductive growth was shortened, and there was less time for the roots to transport nutrients and water to the shoots at later growth stages. The growth and elongation of roots and the photosynthetic capacity and adaptability of leaves to AWD were enhanced under N2. W2N2 treatment delayed root senescence, maintained leaf photosynthesis, optimized the crop growth rate from the full heading to maturity stages, and improved WUE. Hence, the optimal value of rice was obtained at the irrigation water amount and topdressing fertilizer ratio of 41.40–50.34 × 10^2^ and 31.20–34.83 kg ha^–1^, respectively.

## Data Availability Statement

The raw data supporting the conclusions of this article will be made available by the authors, without undue reservation.

## Author Contributions

ZW: data curation and writing-original draft preparation. YJ: conceptualization, methodology, and reviewing the manuscript. JF and ZQ: investigation. XW and HX: software. DZ and JGW: visualization, methodology, and supervision. JW, HL, HLZ, and LY: validation verification and resources. HWZ: conceptualization, methodology, and project administration. All authors contributed to the article and approved the submitted version.

## Conflict of Interest

JW was employed by Bei Da Huang Kenfeng Seed Co., Ltd. The remaining authors declare that the research was conducted in the absence of any commercial or financial relationships that could be construed as a potential conflict of interest.

## Publisher’s Note

All claims expressed in this article are solely those of the authors and do not necessarily represent those of their affiliated organizations, or those of the publisher, the editors and the reviewers. Any product that may be evaluated in this article, or claim that may be made by its manufacturer, is not guaranteed or endorsed by the publisher.
