# Supplementary material for: An Analysis Based on Japonica Rice Root Characteristics and Crop Growth Under the Interaction of Irrigation and Nitrogen Methods
Source: Front Plant Sci. 2022 Jun 28;13:890983. doi: 10.3389/fpls.2022.890983 (PMC9277566; doi:10.3389/fpls.2022.890983)
Supplement: Supplementary file 2 [file Table_2.doc]

**Table S1**

Experiment of nitrogen fertilizer application methods. (kg ha^-1^)

| Treatment | Total pure nitrogen | Basal fertilizer | | | Tillering  fertilizer | Panicle  fertilizer | Grain  fertilizer |
| --- | --- | --- | --- | --- | --- | --- | --- |
|  |  | Urea | Diammonium phosphate | Potassium  sulphate | Urea | Urea | Urea |
| N1 | 150 | 156.5 | 100 | 100 | 97.8 | 32.6 | 0 |
| N2 | 150 | 123.9 | 100 | 100 | 97.8 | 32.6 | 32.6 |
| N3 | 150 | 91.3 | 100 | 100 | 97.8 | 65.2 | 32.6 |
