# Supplementary figures and images for: An Analysis Based on Japonica Rice Root Characteristics and Crop Growth Under the Interaction of Irrigation and Nitrogen Methods
Source: Front Plant Sci. 2022 Jun 28;13:890983. doi: 10.3389/fpls.2022.890983 (PMC9277566; doi:10.3389/fpls.2022.890983)

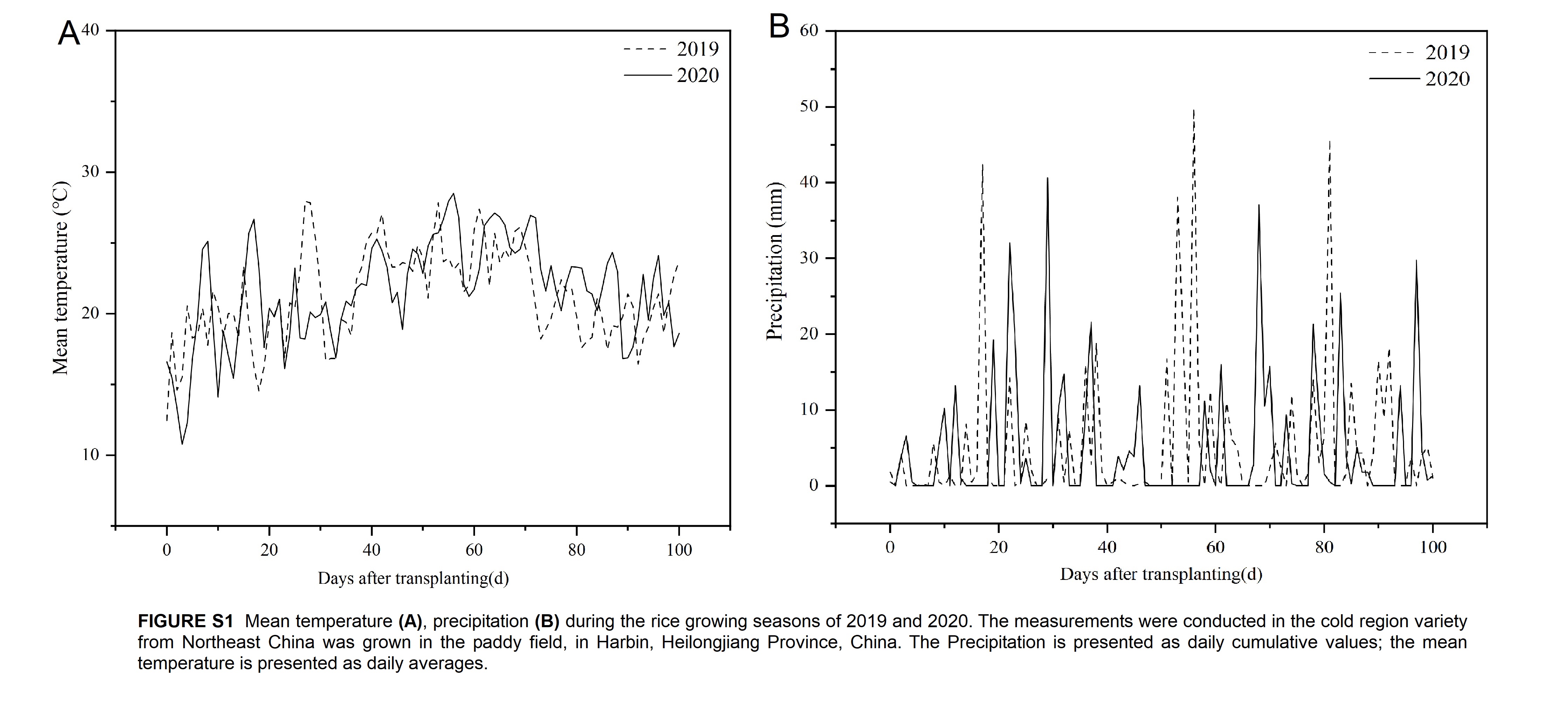

Supplement: Supplementary file 3 [file Image_1.jpg]

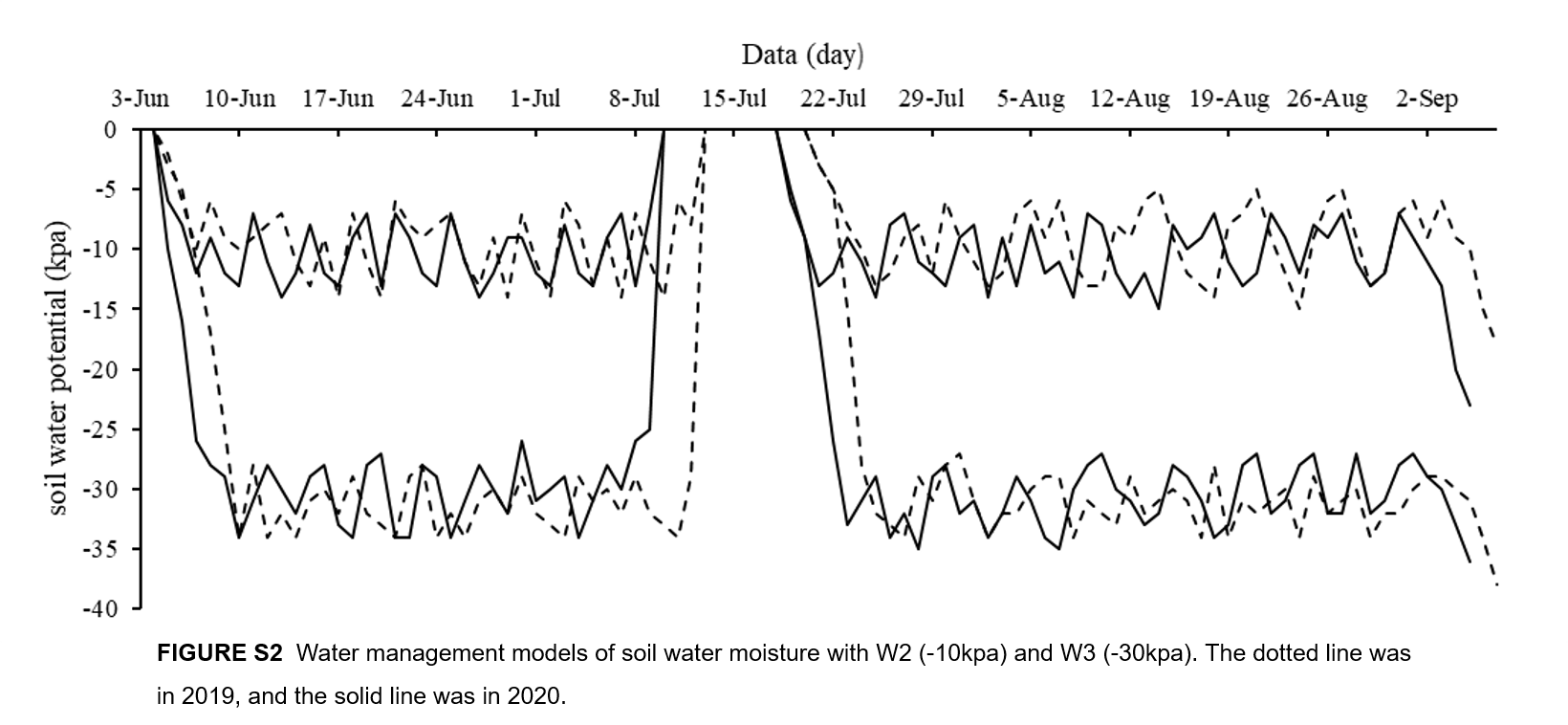

Supplement: Supplementary file 4 [file Image_2.png]
